# The complete chloroplast genome sequence of *Alpinia oxyphylla* Miq. and comparison analysis within the Zingiberaceae family

**DOI:** 10.1371/journal.pone.0218817

**Published:** 2019-06-24

**Authors:** Bingmiao Gao, Lin Yuan, Tianle Tang, Jie Hou, Kun Pan, Na Wei

**Affiliations:** 1 Hainan Provincial Key Laboratory of R&D on Tropical Herbs, Hainan Medical University, Haikou, China; 2 School of Pharmacy, Hainan Medical University, Haikou, China; 3 Environmental Science, School of Tropical and Laboratory Medicine, Hainan Medical University, Haikou, China; National Cheng Kung University, TAIWAN

## Abstract

*Alpinia oxyphylla Miq*. (*A*. *oxyphylla*) is an important edible and traditional herbal medicine. In this study, the complete chloroplast genome of *A*. *oxyphylla* was sequenced, analysed, and compared to five species in the Zingiberaceae family. The size of the *A*. *oxyphylla* chloroplast genome was 161351 bp, which consisted of a large single-copy (LSC, 87248 bp) and small single-copy (SSC, 16175 bp) region separated by a pair of inverted repeats (IRa and IRb, 28964 bp each). The genome encoded 132 unique genes, including 87 protein-coding genes, 37 tRNAs and four rRNAs. The GC content of the genome was 36.17%. A total of 53 simple sequence repeats (SSRs) and 80 long repeats were identified in the *A*. *oxyphylla* chloroplast genome. The chloroplast genome of *A*. *oxyphylla* shared the highest sequence similarity of >90% with the chloroplast genome of *A*. *zerumbet*, and six chloroplast genomes in the Zingiberaceae family were compared by using CGView Comparison Tool (CCT). According to the phylogenetic tree, the Zingiberaceae family is divided into two categories, which coincide with the classification of the characteristics of sun-like and shade-like in plants. Our results reveal the phototrophic component of NADH-dehydrogenase (ndhB and ndhC), photosystem II (psbZ) and ATP synthase (atpE, atpF) exhibit adaptive evolution under different environments, and the strength of light is an important trigger for the adaptations at the chloroplast level.

## Introduction

*Alpinia oxyphylla Miq*. (*A*. *oxyphylla*) is an important edible and traditional Chinese herbal medicine that originates in Hainan and is widely cultivated in southern China [1]. The fruits of *A*. *oxyphylla* have been used as valuable medicines that have a long clinical history and are often used as condiment food in China [2,3]. Numerous studies have reported that *A*. *oxyphylla* is rich in flavonoids, sesquiterpenes, diterpenes, and diarylheptanoids, which have many pharmacological effects, such as improved memory, anti-oxidation, anti-inflammatory, neuroprotective and anticancer[4–8].

Chloroplasts are small organelles inside the cells of plants that contain photosynthetic machinery and produce essential energy for plants [9]. Chloroplasts have their own genetic systems, which consist of a closed circular DNA molecule [9,10]. In recent years, chloroplast genomes have been commonly used for the identification and phyletic evolution analysis of species because of their conserved gene sequences and important role in plants [11]. With the development of high-throughput DNA sequencing technologies, there has been an explosion in the number of available chloroplast genome sequences. However, the chloroplast genome sequences of medicinal plants still require further study. To date, five species of the Zingiberaceae family plant chloroplast genome have been reported, namely, *Alpinia zerumbet*, *Amomum kravanh*, *Curcuma roscoeana*, *Curcuma flaviflora* and *Zingiber spectabile* [12–15]. *A*. *oxyphylla* chloroplast genome sequences have not yet been reported, which has seriously hindered the development of genetic diversity and breeding of *Alpinia* plants. Therefore, it is highly important and essential to study the phylogeny and evolution of Zingiberaceae plants [16].

In this study, we reported the complete chloroplast genome sequence of *A*. *oxyphylla*, including a description of its general features, IR contraction and expansion, codon usage and analysis of SSRs and long repeats. In addition, six chloroplast genome sequences in the Zingiberaceae family were compared by using CGView Comparison Tool (CCT). Moreover, we constructed a phylogenetic tree of the Zingiberales, which provides basic genetic information on the genetic diversity and breeding of *Alpinia* plants.

## Materials and methods

### Ethical statement

No specific permits were required for the collection of specimens for this study.

### Plant material, DNA extraction, and sequencing

Fresh *A*. *oxyphylla* leaves were collected from cultivated fields in Hainan Province, China. Total genomic DNA was extracted from 100 mg of fresh leaves using the Plant Genomic DNA Kit with a standard protocol (Tiangen, Beijing, China). Purified genomic DNA was quantified with a Nanodrop 2000 spectrometer (Thermo Fisher Scientific, Wilmington, USA). Normalized genomic DNA was used to generate a 500 bp (insert size) paired-end library, following the Illumina HiSeq4000 standard protocol. Approximately 2 G raw data were generated with read lengths of 150 bp, and the chloroplast genome sequencing depth was nearly 60×.

### Chloroplast genome assembly and annotation

First, Illumina paired-end reads were filtered on the basis of quality values, and the low-quality reads were trimmed. The remaining clean reads were used for assembly with SOAPdenovo2 (http://soap.genomics.org.cn/soapdenovo.html) on the basis of overlapping and paired-end relationships. Next, all clean reads were mapped onto the assembled contigs to obtain a complete chloroplast genome sequence. Genome confirmation was indispensable to perform after assembly. Finally, the paired-end clean reads were mapped onto the assembled genome with 100% coverage, and the insert-size matched the information of the sequenced library.

Annotation was performed using the online program Dual Organellar GenoMe Annotator (DOGMA) [17]. To prove the correctness of gene and exon boundaries, putative gene and protein sequences were BLAST searched in the Nt and Nr databases. The tRNA genes of *A*. *oxyphylla* were further verified using the online tRNAscan-SE and tRNADB-CE search servers [18–20]. The map of the circular *A*. *oxyphylla* chloroplast genome was drawn through Organellar Genome DRAW (OGDRAW v1.2) [21].

### Genome structure analyses and genome comparison

The distribution of codon usage was detected by the software CodonW (University of Texas, Houston, TX, USA) with the relative synonymous codon usage (RSCU) ratio [22]. The mVISTA program in Shuffle-LAGAN mode was applied to compare the *A*. *oxyphylla* chloroplast genome with five other chloroplast genomes. The boundaries between the IR and SC regions of *A*. *oxyphylla* and five other Zingiberaceae species were compared and analysed. The visualization of codon usage in the form of heatmaps of 17 species of Zingiberales and a histogram were conducted with R language with an RSCU value.

### Repeat sequence analyses

Repeat sequences in chloroplast genomes were detected by the REPuter program [23], including forward, reverse, palindrome, and complement sequences in the chloroplast genome of *A*. *oxyphylla*. The length and identity of the repeats were limited to ≥ 30 bp and >90%, respectively [24]. The SSRs were searched using MISA [25], with the following repeat threshold settings: 10 repeats for mono-nucleotide, 5 repeats for di-, 4 repeats for tri-nucleotide, and 3 repeats for tetra- and penta-nucleotide SSRs [26].

### CCT map

The *A*. *oxyphylla* chloroplast genome was compared with other available chloroplast genomes of Zingiberales by using CCT [27]. Genes were assigned by Clusters of Orthologous Groups, and BLAST was used to align other genomes to that of *A*. *oxyphylla*. The visualization of the circular map was conducted by CCT. AT distributions were measured on the basis of AT skewed using the equation: AT-skew = (A−T)/(A+T).

### Phylogenetic analysis

Concatenated alignments of 17 chloroplast genome sequences were performed using MUSCLE v.3.8.31. The phylogenetic analysis was carried out using the ML method with RAxML8.1, and the trees were visualized and annotated using the tree viewer of MEGA6 [28]. Statistical supports were assessed with 1000 bootstrap pseudo-replicates.

### Positive selection analysis of protein sequence

To investigate the evolutionary process of light adaptation of Zingiberaceae plants, we calculated the Nonsynonymous (Ka), Synonymous (Ks) and Ka/Ks ratios of protein coding genes associated with the photosystem using KaKs_Calculator 2.0 [29].

## Results and discussion

### General features of the *A*. *Oxyphylla* chloroplast genome

**Fig 1:**
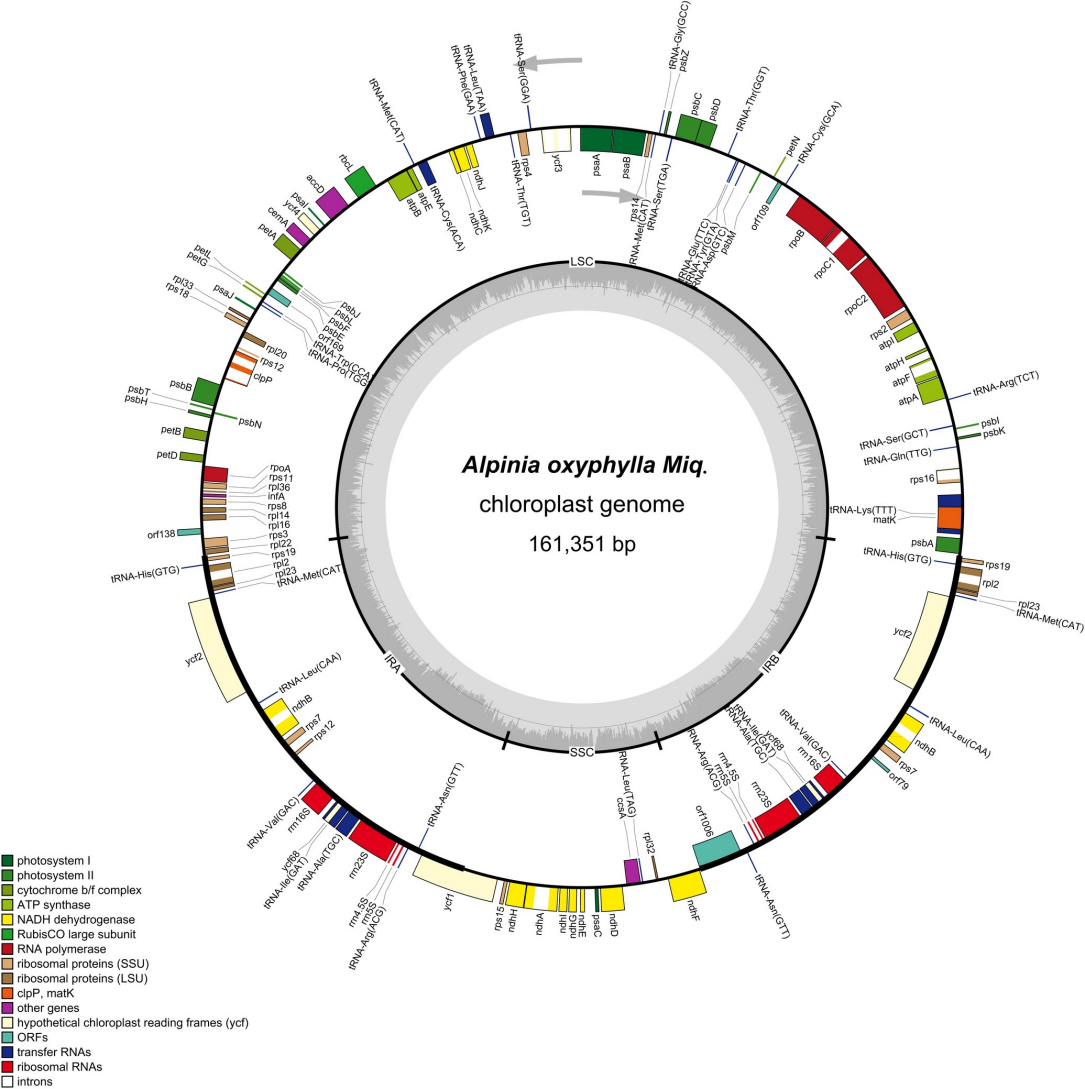
Gene map of the complete chloroplast genome of *A*. *oxyphylla*. Genes on the outside of the circle are transcribed clockwise, while those inside are counterclockwise. Genes belonging to different functional groups are colour-coded. The darker and lighter grey in the inner circle correspond to GC and AT content, respectively.

The complete chloroplast genome of *A*. *oxyphylla* (GenBank Accession Number: KY985237) has a typical quadripartite structure and is a circular molecule 161,351 bp in size (Fig 1 and Table 1). The genome contains a small single-copy (SSC) region of 16175 bp and a large single-copy (LSC) region of 87248 bp, separated by a pair of inverted repeats (IRa and IRb) of 28964 bp each (Fig 1 and Table 1). The GC content of the *A*. *oxyphylla* chloroplast genome is 36.17%, which is similar to other chloroplast genomes previously reported [14,15,30]. The genome consists of 132 genes, including 87 distinct protein-coding genes, four distinct rRNA genes and 37 distinct tRNA genes, 21 of which were duplicated in the IR regions, 12 in the SSC region and 84 in the LSC region (Table 1).

**Table 1:** Gene contents in the complete chloroplast genome of *A*. *oxyphylla*.

Category of genes	Group of gene	Name of gene
**Self-replication**	Small subunit of ribosome (SSU)	rps2, rps3, rps4, rps7*, rps8, rps11, rps12, rps14, rps15, rps16, rps18, rps19*
	Large subunit of ribosome (LSU)	rpl2*, rpl14, rpl16, rpl20, rpl22, rpl23*, rpl32, rpl33, rpl36
	DNA-dependent RNA polymerase	rpoA, rpoB, rpoC1, rpoC2
	Ribosomal RNA genes (rRNA)	rrn16S*, rrn5S*, rrn23S*, rrn4.5S*
	Translation initiation factor	infA
	Transfer RNA genes (tRNA)	trnA-TGC*, trnC-GCA, trnC-ACA, trnD-GTC, trnE-TTC, trnF-GAA, trnG-GCC, trnH-GTG*, trnI-GAT*, trnK-TTT, trnL-TAA, trnL-CAA*, trnL-TAG, trnM-CAT*, trnN-GTT*, trnP-TGG, trnQ-TTG, trnR-TCT, trnR-ACG*, trnS-GCT*, trnS-TGA, trnS-GGA, trnT-GGT, trnT-TGT, trnV-GAC*, trnW-CCA, trnY-GTA
**Genes for photosynthesis**	Subunits of NADH dehydrogenase	ndhA, ndhB*, ndhC, ndhD, ndhE, ndhF, ndhG, ndhH, ndhI, ndhJ, ndhK
	Large subunit of Rubisco	rbcL
	Subunits of photosystem II	psbA, psbB, psbC, psbD, psbE, psbF, psbH, psbI, psbJ, psbK, psbL, psbM, psbN, psbT, psbZ
	Subunits of photosystem I	psaA, psaB, psaC, psaI, psaJ
	Subunits of ATP synthase	atpA, atpB, atpE, atpF, atpH, atpI
	Subunits of cytochrome	petA, petB, petD, petG, petL, petN
	Photosystem I assembly	ycf3, ycf4
**Other genes**	Envelope membrane protein	cemA
	C-type cytochrome synthesis gene	ccsA
	Subunit of acetyl-CoA	accD
	Protease	clpP
	Maturase	matK
**Unknown**	Conserved open reading frames	orf109, orf138, orf169
**Pseudogenes**		ycf1, ycf2, ycf68*, orf79, orf1006

Note

* indicates a duplicated gene.

The size of the *A*. *oxyphylla* chloroplast genome was similar to those of five Zingiberaceae family species (Table 2). The size of the *A*. *kravanh* chloroplast genome (162766 bp) is the longest, and the *Z*. *spectabile* chloroplast genome (155890 bp) is the shortest. Interestingly, the SSC region (15,390 bp) of *A*. *kravanh* is the shortest, whereas the SSC region (18611 bp) of the *Z*. *spectabile* chloroplast genome is the longest. Five of the chloroplast genomes contain 132 genes, excluding only *A*. *kravanh* (135 genes). As shown in Table 2, the *C*. *roscoeana* chloroplast genome has the highest GC content (36.33%), while the *A*. *kravanh* chloroplast genome has the lowest GC content (31.3%). In addition, 87 protein genes were identified in *A*. *oxyphylla*, 80 were identified in *A*. *kravanh*, and 86 were identified in the other four species. Four conserved rRNAs were identified in every species. The *A*. *oxyphylla* chloroplast genome encodes 37 types of tRNAs, *A*. *zerumbet*, *C*. *roscoeana*, *C*. *flaviflora* and *Z*. *spectabile* encode 38, whereas *A*. *kravanh* encodes 30 (Table 2).

**Table 2:** Comparison of the general features of the six Zingiberaceae chloroplast genomes.

Genome feature	*A*. *oxyphylla*	*A*. *zerumbet*	*A*. *kravanh*	*C*. *roscoeana*	*C*. *flaviflora*	*Z*. *spectabile*
GenBank	KY985237	JX088668	MF991963.1	KF601574	NC_028729.1	NC_020363.1
**Size (bp)**	161351	159773	162766	159512	160478	155890
**LSC (bp)**	87248	87644	87728	87015	88008	85983
**SSC (bp)**	16175	18295	15390	18531	18570	18611
**IR (bp)**	28964	26917	29824	26983	26950	25554
**Total genes**	132	132	135	132	132	132
**Protein genes**	87	86	80	86	86	86
**tRNA genes**	37	38	30	38	38	38
**rRNA genes**	4	4	4	4	4	4
**GC (%)**	36.17%	36.27%	31.3%	36.33%	36.30%	36.29%

### IR contraction and expansion

**Fig 2:**
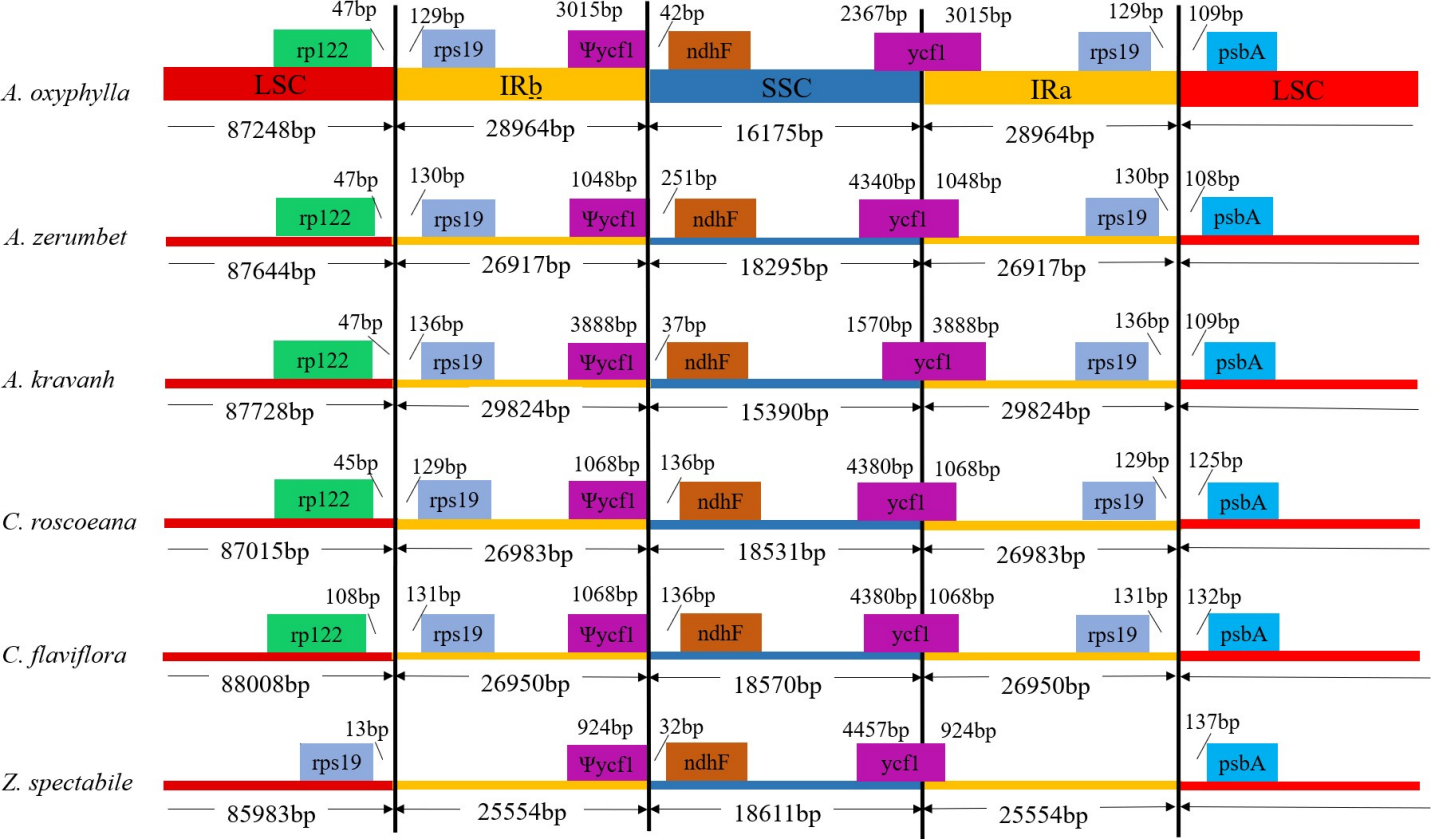
Comparison of the borders of the LSC, SSC and IR regions among the six chloroplast genomes. Ψ, pseudogenes.

The contraction and expansion of the IR region are common evolutionary events and are considered the major reasons for size differences in different chloroplast genomes, which is best for studying the phylogeny and the chloroplast genome evolution history of early land plant lineages [31,32]. In the *A*. *oxyphylla* chloroplast genomes, the boundary of IR/LSC extended into the rps19 gene, and 129 bp of rps19 extended into the IR region; the boundary of IR/SSC extended into the ndhF gene, and 42 bp of ndhF extended into the IR region; the boundary of IRb/SSC extended into 3015 bp of ycf1; and the boundary of IRb/LSC and IRa/LSC extended into the rpl22 and psbA genes, respectively. In this study, a detailed comparison of the borders among the IR, LSC and SSC regions among the six Zingiberaceae chloroplast genomes is presented in Fig 2. The pseudogene ycf1 is often used to study genetic variation in the chloroplast genome in higher plants [32], and the length ranges from 924 to 3888 bp in the six comparable chloroplast genomes. The ndhF gene was 32, 37, 42, 136,136 and 251 bp from the IRb and SSC border in *Z*. *spectabile*, *A*. *kravanh*, *A*. *oxyphylla*, *C*. *roscoeana*, *C*. *flaviflora* and *A*. *zerumbet*, respectively. The rps19 gene is usually one of the most abundant transcripts among the plant chloroplast genome, including five comparable chloroplasts. However, the rps19 gene was completely located in the LSC region in the *Z*. *spectabile* chloroplast genome. Our results suggest that the IR/LSC boundary might be conversed among the chloroplast genomes of closely related family species, but greater diversity also occurs between relatively distantly related family species, such as *Z*. *spectabile* [33,34].

### Codon usage

**Fig 3:**
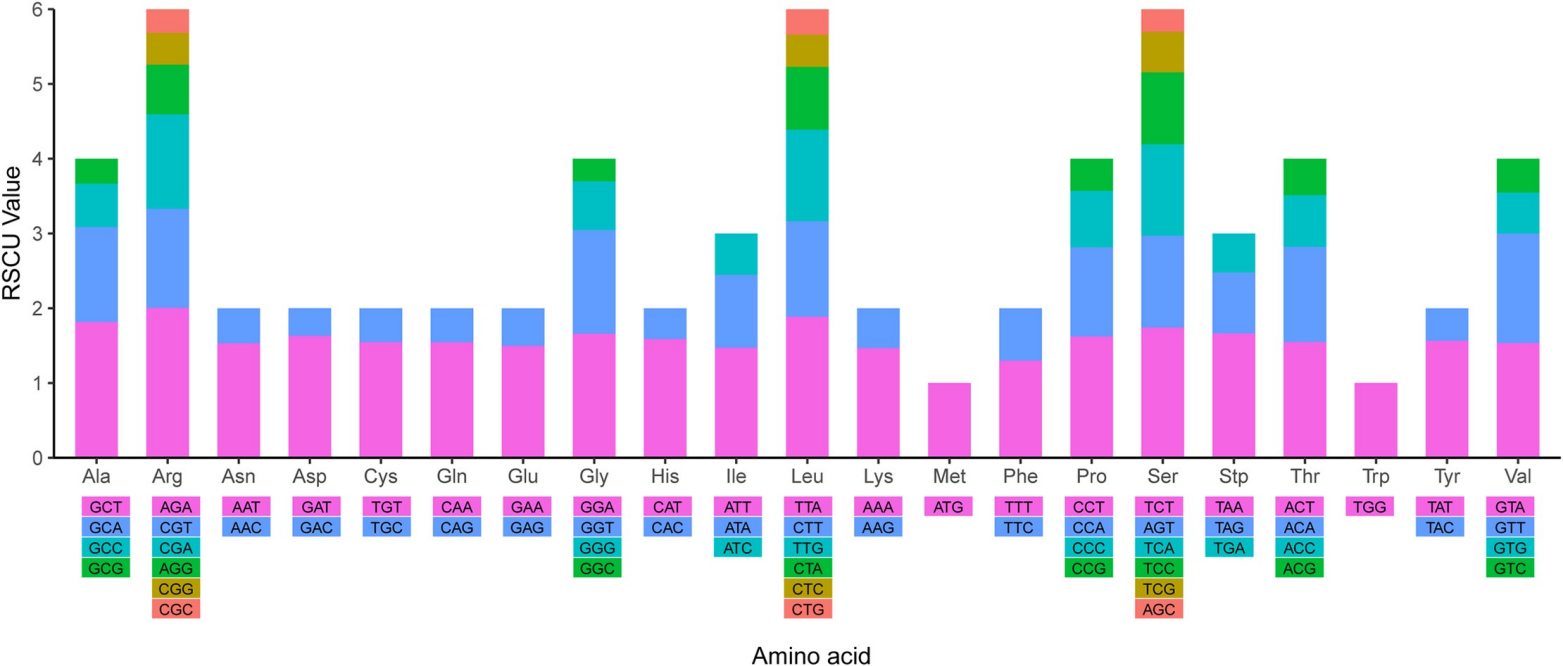
Codon content of 20 amino acids and stop codons in all protein-coding genes of the *A*. *oxyphylla* chloroplast genome. The colour of the histogram corresponds to the colour of codons.

**Fig 4:**
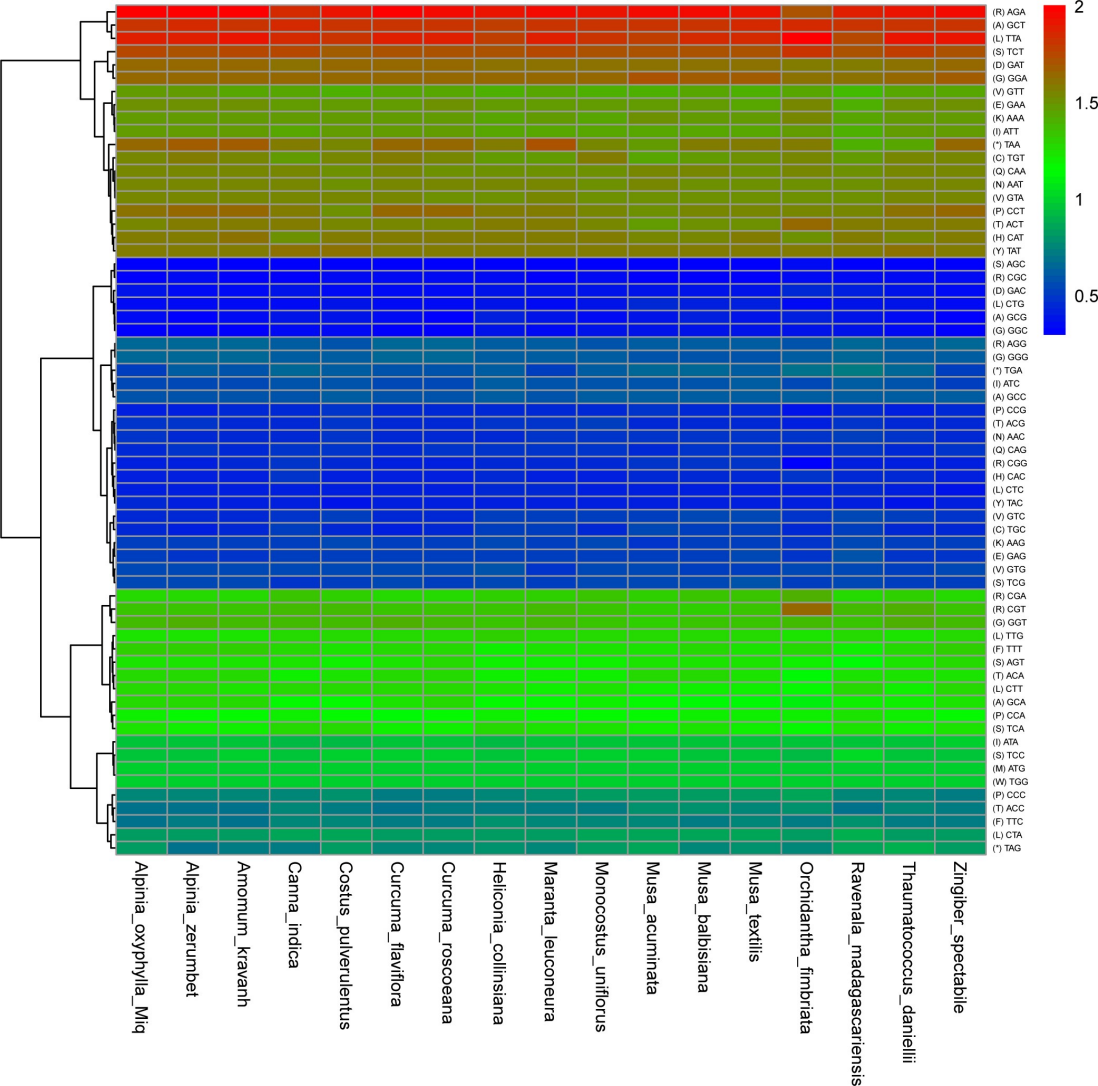
Heatmap analysis for codon distribution of all protein-coding genes of all considered species. Colour key: higher red values indicate higher RSCU values, and lower blue values indicate lower RSCU values.

The standard ATG codon is typically the start codon for most protein-coding genes. However, ATA and ATC are also used as alternatives to ATG as the initiation codon under certain circumstances [35]. The initiation codon ATG of three genes was replaced among the *A*. *oxyphylla* chloroplast protein-coding genes, which were ATC for rps12 and orf79 and ATA for rp12(S1 Table). The codon usage frequency and relative synonymous codon usage (RSCU) were analysed based on sequences of 87 distinct protein-coding genes in the *A*. *oxyphylla* chloroplast genome (Fig 3). The high RSCU value was probably attributed to the function of the amino acid or the structure of the peptide to avoid error in transcription [35]. As shown in Fig 4, the result of the distributions and the visualization of codon usage in the form of heatmaps of 17 species of Zingiberales suggested that approximately one-third of the codons were not frequently used. These codons are shown in blue, which indicates an RSCU value of <1 and weak codon bias. The results showed evident codon use preferences for *A*. *oxyphylla*, among which AGA, TTA, GCT, TCT, and AGA were used most frequently (Fig 4). Approximately two-thirds of all codons of *A*. *oxyphylla* that had high RSCU values showed a high A/T preference in the third codon. This phenomenon is common in the chloroplast genomes of higher plants [36].

### Analysis of SSRs and repeats

**Fig 5:**
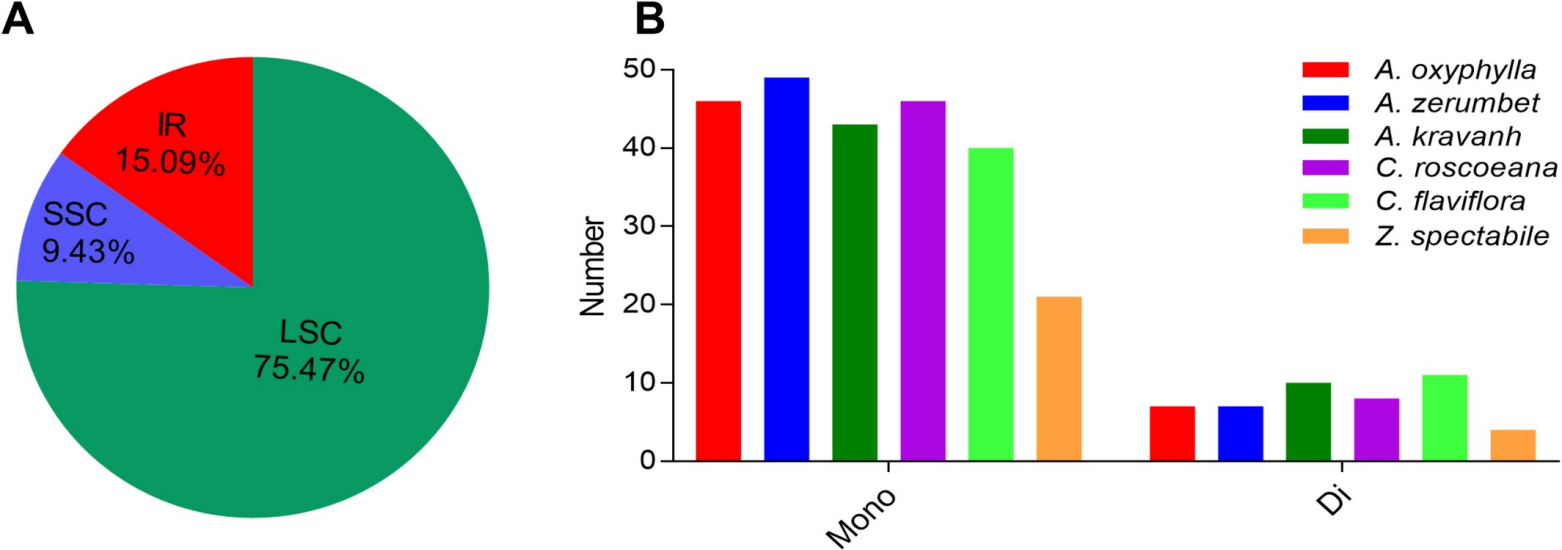
SSR analysis of the six Zingiberaceae chloroplast genomes. (**A**) Presence of SSRs in the LSC, SSC and IR regions (*A*. *oxyphylla*); (**B**) Presence of polymers in the chloroplast genome of *A*. *oxyphylla*, *A*. *zerumbet*, *A*. *kravanh*, *C*. *roscoeana*, *C*. *flaviflora*, and *Z*. *spectabile*.

Simple sequence repeats (SSRs), also known as microsatellites, are a group of tandem repeated sequences, generally ranging in length from 1–6 or more base pairs and are widely distributed in chloroplast genomes [37]. A total of 53 SSRs were detected from the *A*. *oxyphylla* chloroplast genome, including 46 mono- and 7 di-nucleotide SSRs, which were located in the LSC region (75.47%), IR region (15.09%) and SSC region (9.43%), respectively (Fig 5 and S2 Table). Furthermore, the distribution pattern and number of SSRs among the six Zingiberaceae chloroplast genomes (*A*. *oxyphylla*, *A*. *kravanh*, *A*. *zerumbet*, *C*. *flaviflora*, *C*. *roscoeana*, and *Z*. *spectabile*) were compared, and the results suggested that there was little difference in the distribution pattern and number of SSRs among the six chloroplast genomes (Fig 5). In addition, a total of 80 repeats were detected in the *A*. *oxyphylla* chloroplast genome, including 4 complement, 35 forward (direct) repeats, 36 palindrome (inverted) repeats and 14 reverse repeats (S3 Table). Fifty-seven repeats were located in the intergenic spacers (IGS) regions, 19 repeats were located in coding sequence (CDS) regions, and 4 repeats were located in intron regions. These SSRs and repeats can be made into lineage-specific markers, which can provide genetic diversity analysis for *A*. *oxyphylla* and its related species [38,39].

### CCT map

**Fig 6:**
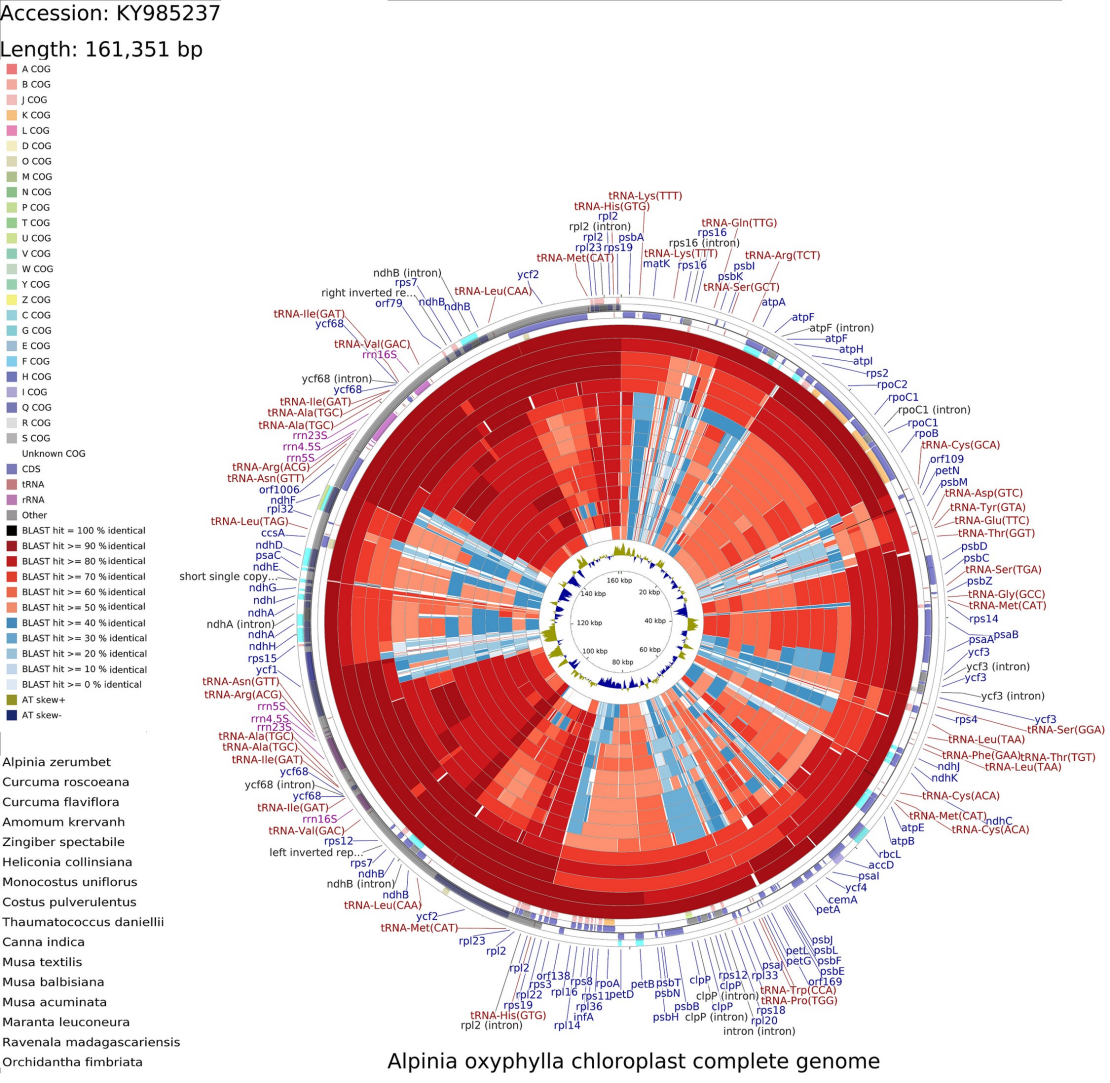
CCT map comparison of 17 chloroplast genomes of *A*. *oxyphylla* to *Orchidantha fimbriata*. The four outer rings are the protein-coding gene positions based on the *A*. *oxyphylla* chloroplast genome. The innermost ring displays AT skew in *A*. *oxyphylla*. The remaining rings display regions of similarity among the 17 compared chloroplast genomes.

**Fig 7:**
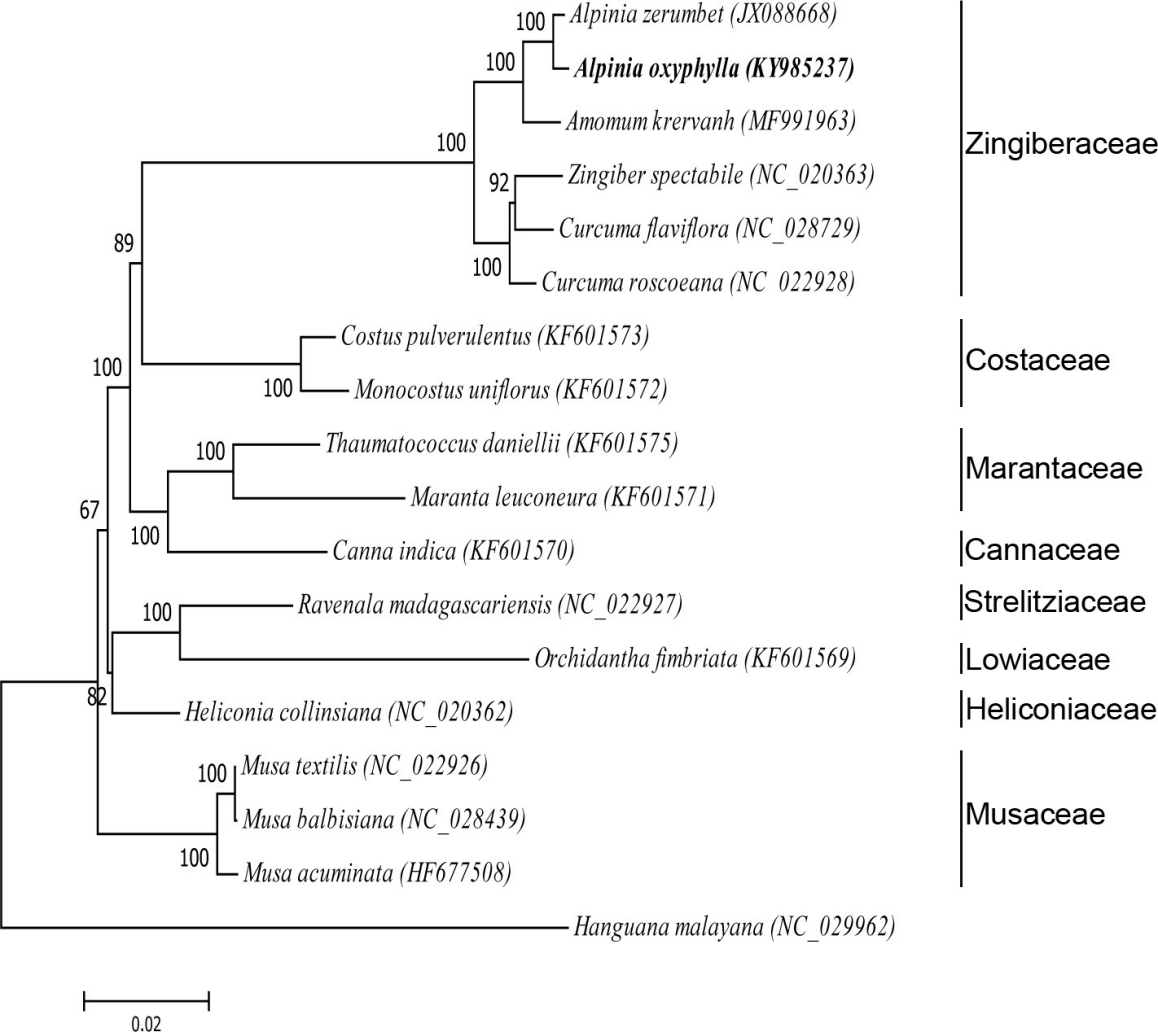
The maximum parsimony (MP) phylogenetic tree based on chloroplast genome sequences. The numbers in each node were tested by bootstrap analysis with 1000 replicates.

CCT is a package for visually comparing circular bacterial, plasmid, chloroplast, or mitochondrial genome sequences [40]. The *A*. *oxyphylla* chloroplast genome was compared with 16 previously reported chloroplast genomes of Zingiberales by using CCT (Fig 6). The results showed that the highest sequence similarity (>90%) was between the chloroplast genomes of *A*. *oxyphylla* and *A*. *zerumbet*, which was consistent with the result of the phylogenetic analysis (Figs 6 and 7). The most similar region appears in the IR region, and diversity exists in the LSC and SSC regions among 17 chloroplast genomes. This evolutionary feature of the chloroplast genome has also been reported in other plants [41].

### Phylogenetic analysis

Complete chloroplast genomes contain a substantial amount of phylogenetic information, which has been used for phylogenetic analysis of deep relationships among the primary clades of Zingiberales [14,16,34,42]. To identify the evolutionary position of *A*. *oxyphylla* within Zingiberales, an improved resolution of phylogenetic relationships was achieved by using these complete chloroplast genome sequences of 17 Zingiberales species. The maximum likelihood (ML) bootstrap values had values of 100% bootstrap support for the Zingiberaceae family except the node of *Z*. *spectabile* and *C*. *flaviflora* (92%) (Fig 7). The *A*. *oxyphylla* chloroplast genome was closely related to *A*. *zerumbet* and *A*. *kravanh*, which then formed a cluster with *Z*. *spectabile*, *C*. *roscoeana*, and *C*. *flaviflora* with 100% bootstrap supports. In addition, the four ginger families form a well-supported clade within which the families Zingiberaceae and Costaceae, Marantaceae and Cannaceae are sisters. Therefore, the results are expected to be useful in resolving the deeper branches of the phylogenetic tree and will help expand the understanding of the evolutionary history of Zingiberaceae, particularly regarding the role of *A*. *oxyphylla* in plant systematics and evolution.

### Adaptive evolution analysis

**Fig 8:**
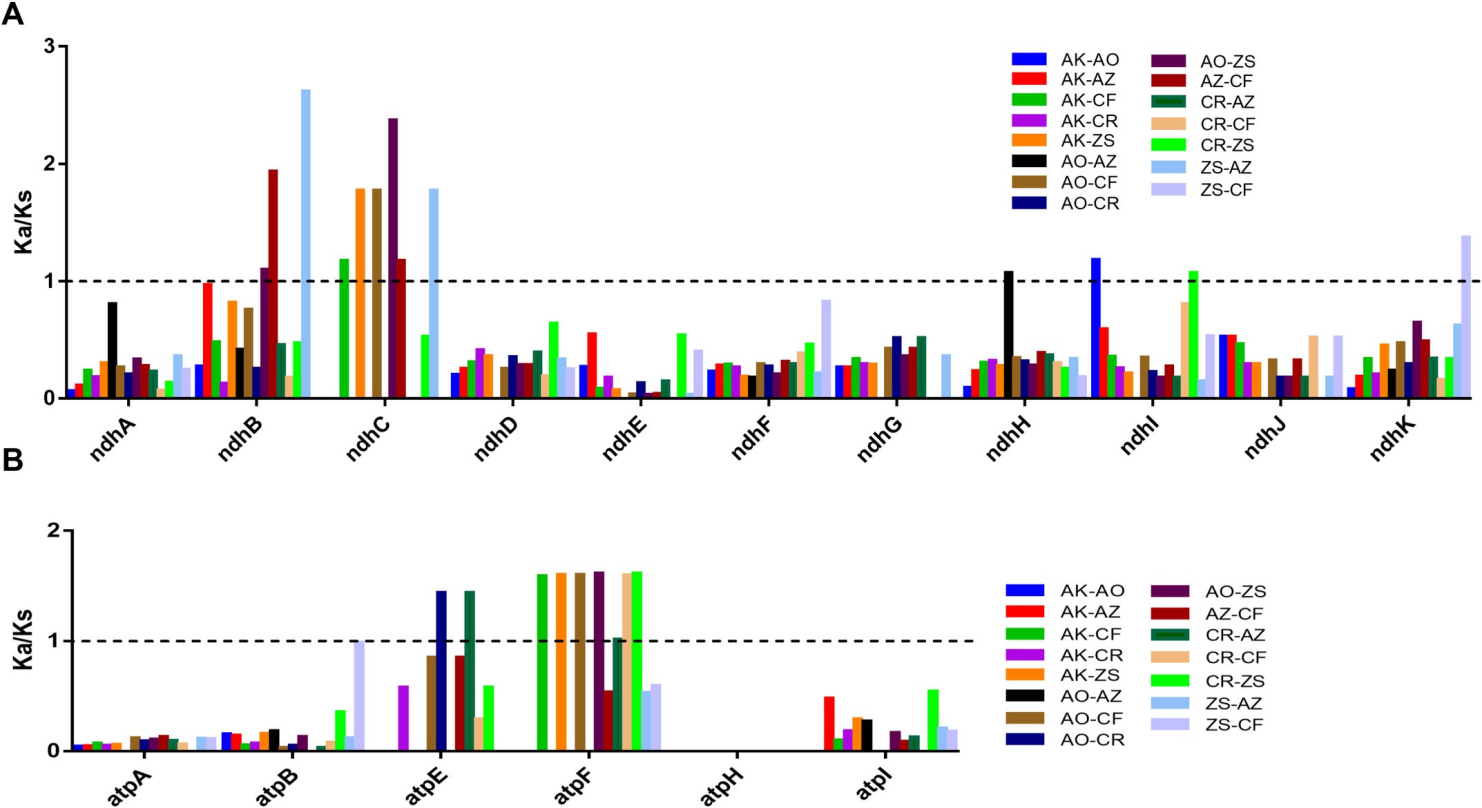
**Ka/Ks ratios of NADH-dehydrogenase (A) and ATP synthase (B) coding genes.** Ka, nonsynonymous; Ks, synonymous; AO, *A*. *Oxyphylla*; AK, *A*. *kravanh*; AZ, *A*. *Zerumbet*; CR, *C*. *Roscoeana*; CF, *C*. *Flaviflora*; ZS, *Z*. *Spectabile*.

The Zingiberaceae family is divided into two main categories, *A*. *oxyphylla*, *A*. *kravanh* and *A*. *zerumbet* clustered into one branch, *C*. *roscoeana*, *C*. *flaviflora* and *Z*. *spectabile* clustered into another (Fig 7). Interesting, the former are typical shade-loving plants whose natural habitats are shade forests of South China, and the latter is sun-loving plants. To investigate the evolutionary process of light adaptation of Zingiberaceae plants, we calculated Ka/Ks ratios of NADH-dehydrogenase, photosystem I, photosystem II, cytochrome b/f complex and ATP synthase coding genes associated with the photosystem. The Ka/Ks is a powerful approach for measuring selective pressure at the protein-coding level. The genes with positive selection played key roles in the adaptation to diverse environment [43]. As a result, NADH-dehydrogenase (ndhB, ndhC, ndhH, ndhI, ndhK), photosystem II (psbZ) and ATP synthase (atpE, atpF) coding genes with Ka/Ks > 1 were detected, indicating that these genes are undergoing positive selection (Fig 8 and S4 Table). Moreover, the Ka/Ks ratios of the gene ndhC and atpF in four pairwise comparisons of *A*. *kravanh*—*C*. *flaviflora*, *A*. *kravanh*—*Z*. *spectabile*, *A*. *oxyphylla*—*C*. *flaviflora* and *A*. *oxyphylla*—*Z*. *spectabile* were both > 1, indicating that these two genes are critical in adapting to light (Fig 8). Our results reveal the phototrophic component of NADH-dehydrogenase (ndhB and ndhC), photosystem II (psbZ) and ATP synthase (atpE, atpF) exhibit adaptive evolution under different environments, and the strength of light is an important trigger for the adaptations at the chloroplast level.

## Conclusions

The complete chloroplast sequence of *A*. *oxyphylla* contains LSC, SSC, and IR regions. A total of 53 SSRs and 80 repeats were identified in the *A*. *oxyphylla* chloroplast genome. The CCT analytical results indicated that the *A*. *oxyphylla* chloroplast genome shared the highest sequence similarity with *A*. *zerumbet*. Phylogenetic trees strongly supported classification of the characteristics of sun-like and shade-like in Zingiberaceae plants. Moreover, the results will help expand the understanding of the evolutionary history of Zingiberaceae, particularly regarding the phototrophic component of NADH-dehydrogenase (ndhB and ndhC), photosystem II (psbZ) and ATP synthase (atpE, atpF) exhibit adaptive evolution under different environments.

## Supporting information

S1 Table Summary of gene/element features. (XLS)

S2 Table Simple sequence repeats in the *A. oxyphylla* chloroplast genome. (XLS)

S3 Table Long repeat sequences in the *A. oxyphylla* chloroplast genome. (XLS)

S4 Table Ka/Ks ratios of protein coding genes associated with the photosystem. (XLSX)
